# Successful Kidney Transplantation for End-Stage Renal Disease in Marfan's Syndrome

**DOI:** 10.1155/2013/809613

**Published:** 2013-09-04

**Authors:** Makoto Ryosaka, Kazuya Omoto, Taiji Nozaki, Kazuhiko Yoshida, Yugo Sawada, Hajime Hirano, Tomokazu Shimizu, Hideki Ishida, Kazunari Tanabe

**Affiliations:** Department of Urology, Tokyo Women's Medical University, 8-1 Kawada-cho, Shinjuku-ku, Tokyo 162-8666, Japan

## Abstract

Marfan's syndrome is a systemic disorder of the connective tissue caused by mutations in the extracellular matrix protein fibrillin-1, with aortic dissection and aneurysm being its most life-threatening manifestations. Kidney transplantation for end-stage renal disease (ESRD) in patients with Marfan's syndrome has not been reported in the literature, and the rate of the incidence of dissection or aneurysm in the iliac artery is unknown. Here, we present a patient with Marfan's syndrome with ESRD due to severe renal ischemia caused by massive bleeding from thoracoabdominal aortic dissection leading to transplant surgery of a living kidney procured from the patient's mother. After kidney transplantation, the renal function normalized without vascular complications, and stable graft function along with negative results for both microhematuria and proteinuria continued for two years. Also, vascular complication such as aneurysm or dissection of the iliac artery was not observed using ultrasonography during the follow-up period. ESRD patients with Marfan's syndrome might be suitable for kidney transplantation, but long-term and careful observations are needed.

## 1. Introduction

Marfan's syndrome is an autosomal dominant condition with a reported incidence of one in 3,000 to 5,000 individuals [1]. It is caused by mutations in the gene encoding fibrillin-1 (*FBN1*). *FBN1* mutations cause connective tissue disorder in terms of classic ocular, cardiovascular, and musculoskeletal abnormalities. Manifestations include involvement of the lung, skin, and central nervous system. One of the most important clinical problems is the occurrence of thoracoabdominal aortic aneurysm and/or dissection. Most commonly, the dilation of the ascending aorta at the level of the aortic sinus is a cardiovascular manifestation of the disease. Progressive aorticsinusenlargement leading to aortic aneurysm is present in 50%–60% of adults and in 50% of children with Marfan's syndrome. Recent data suggest that Marfan's syndrome is present in 50% of patients presenting with aortic dissection under the age of 40 and accounts for only 2% of dissections in older patients [2, 3]. Because of this, the lifespan of patients with Marfan's syndrome is often shortened. A report in the early 1970s describes that life expectancy for affected individuals was about two-thirds that of individuals without Marfan's syndrome [4]. However, a recent assessment of outcome in Marfan's syndrome describes a nearly normal life expectancy by virtue of aortic replacement and cardiac transplantation. In general, Marfan's syndrome in itself will not cause renal dysfunction. Some of these patients, however, develop ESRD because of aortic dissection, abdominal aortic aneurysm, or/and the operations for them. Enlargement of the distal aorta and its branches is less common than an ascending aortic enlargement. A previous report recommended graft replacement of the aortic segment involved if the expansion rate accelerates to ≧1 cm/year, or when symptoms occur, or the aorta reaches 6.0–6.5 cm in diameter [5]. In tandem with abdominal aortic dissection, renal function is sometimes severely injured by impairment of blood flow to the renal arteries, which induces end-stage renal disease (ESRD).

As far as we can ascertain, the techniques and problems of kidney allograft transplantation in Marfan's syndrome have not been described previously. Here, we report a case of living kidney transplantation in an ESRD patient with Marfan's syndrome.

## 2. Case Report

A pregnant 33-year-old woman with Marfan's syndrome was found to have an abdominal aortic aneurysm approximately 7 × 6 cm in diameter, stretching from the bilateral renal arteries to the common iliac arteries. After Caesarean section, she underwent a composite graft replacement for abdominal aortic aneurysm that bypassed her celiac, superior mesenteric, and bilateral renal arteries with a branched graft. Two weeks later, a thoracoabdominal aortic dissection suddenly occurred. This prompted the emergency replacement of her endovascular stent graft. The replacement of the stent and massive bleeding led to severe ischemic renal injury in both kidneys. Her renal function gradually decreased, and four months later, maintenance hemodialysis was required due to ESRD induced by irreversible ischemic renal damage.

She and her family had been considering a living kidney transplantation ever since she began maintenance hemodialysis. The donor was her 60-year-old mother, because her father had been diagnosed with Marfan's syndrome and had also undergone a composite graft replacement for thoracoabdominal aortic aneurysm. 

For treatment of annuloaortic ectasia and preparation for kidney transplantation, our patient underwent aortic root replacement using the David aortic valve-sparing reimplantation technique two years after the commencement of maintenance hemodialysis. Her cardiac function has been stable since the aortic valve-sparing operation. 

**Figure 1:**
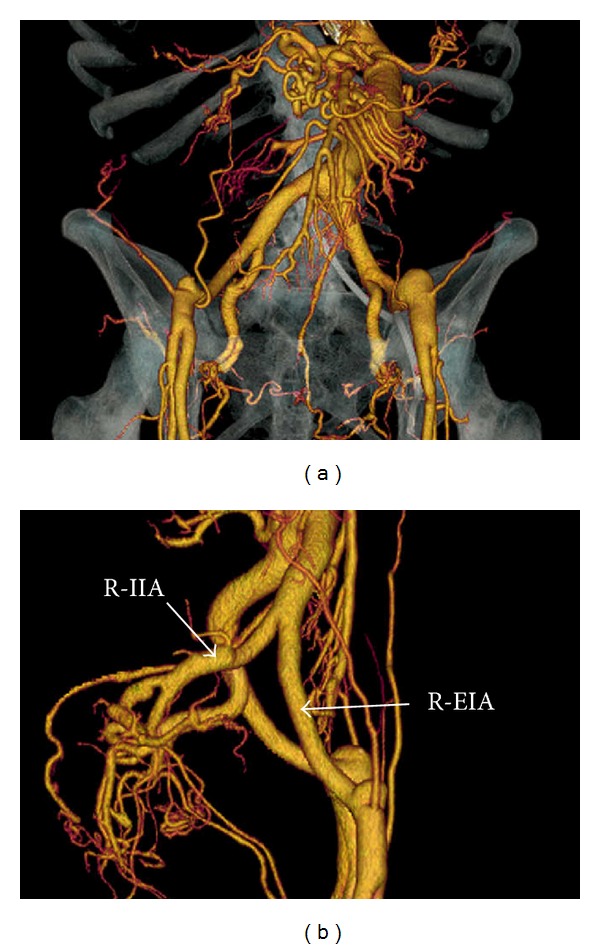
Three-dimensional computed tomography scan demonstrating no dissection or aneurysm in the iliac and external iliac arteries. (a) Anterior to posterior imaging. (b) Right-to left-sided imaging. R-IIA: right internal iliac artery; R-EIA: right external iliac artery.

Although the patient had many anti-human leukocyte antigens (HLA) antibodies due to repeated blood transfusions, donor-specific HLA antibodies were not found. Blood type of donor and recipient were types B and O, respectively. The titer of anti-blood type B antibody was 64 : 1. The preoperative, 3-dimensional CT scan showed that there was no aneurysm or dissection of the iliac arteries (Figure 1). 

**Figure 2:**
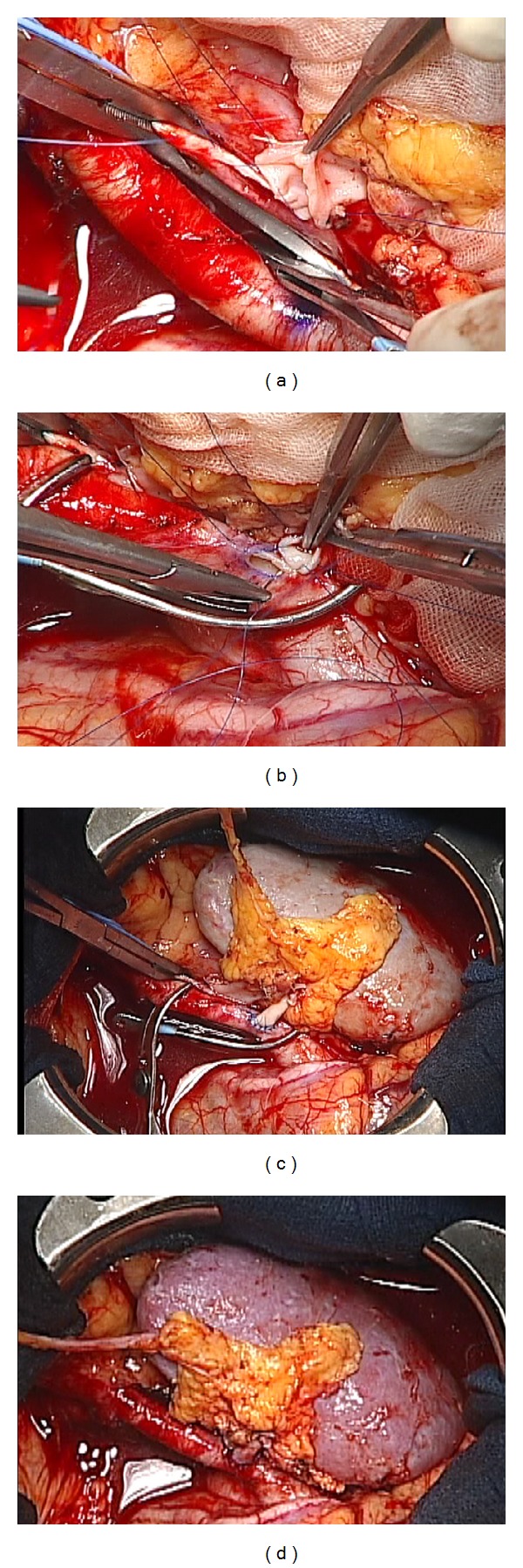
(a) The left renal vein of donor kidney anastomosed to the right external iliac artery end-to-side with a continuous suture. (b) The renal artery anastomosed to the right iliac artery by end-to-side, continuous and interrupted sutures. (c) All anastomoses completed right before the declamping of vessels.(d) There was no bleeding at the anastomoses, and blood flow to the renal artery was stable.

The patient received a living kidney transplantation from her mother 3 years and 5 months after the onset of hemodialysis. The initial immunosuppression consisted of tacrolimus, mycophenolate mofetil, methylprednisolone, basiliximab, and rituximab. Before transplantation, she underwent double filtration plasma pheresis twice to reduce the anti-blood type B antibody. We transplanted a left-sided kidney into the right iliac fossa. Although we had predicted severe adhesion around the external iliac artery and vein because of previous surgery for graft replacement, it was comparatively easy to dissect those vessels. The renal vein was anastomosed to the external iliac vein (Figure 2(a)). The renal artery was then anastomosed to the external iliac artery (Figure 2(b)). We could not anastomose the renal artery to her internal iliac artery end-to-end because the vessel diameters were very different (Figure 2(c)). After the declamping of vessels, a good blood supply was established to the transplanted kidney (Figure 2(d)). Operating time was 234 minutes, and the estimated blood loss was 117 mL. After surgery an ultrasonography showed that there was no dissection or aneurysm of the iliac artery. The serum creatinine level was 1.37 mg/dL at day four after transplantation. Two months after transplantation, we performed a protocol biopsy, which indicated that the specimen showed no signs of rejection. Eight months after transplantation, renal function remained stable, and the serum creatinine level was 1.0 mg/dL. Microhematuria and proteinuria were not detected. In addition, neither dissection nor aneurysm was observed by ultrasonography two years after the kidney transplantation. 

## 3. Discussion

The incidence of acute renal injury after thoracoabdominal aortic aneurysm and aortic dissection repair has been reported to be about 30%, with renal failure that requires hemodialysis occurring in about 3% of patients with or without Marfan's syndrome [6]. In terms of kidney transplantation, a report of successful preservation of threatened renal function by renal autotransplantation, following dissection of the descending aorta, has been published in a patient with Marfan's syndrome [7]. There is also a report of kidney transplantation from a deceased donor with Marfan's syndrome [8]. As far as we know, however, there are no reports in the literature of successful living-related kidney transplantation in a patient with Marfan's syndrome. It seems to be difficult to anastomose blood vessels because of their connective tissue disorder. In the present case, we performed a kidney transplantation in a patient with Marfan's syndrome without any complications. After transplantation, although there was a small hematoma near the upper pole of the transplanted kidney, vascular complications of the kidney graft did not occur, and blood flow in the peripheral areas of the graft was clearly stable. 

Immunosuppressive treatment for patients of Marfan's syndrome needs to be discussed. The use of immunosuppressive therapy in patients with Marfan's syndrome is a matter of controversy, as hypertension induced by calcineurin inhibiters and steroid therapy may reduce the integrity of the venous connective tissue. We do not know whether it is safe to use tacrolimus because, as far as we know, there has been no report of administration of tacrolimus to a patient with Marfan's syndrome. Furthermore, it is reported that long-term use of prednisolone leads to aneurysm [9, 10]. However, some reports suggest that there was no problem in using immunosuppressive drugs (cyclosporine, mycophenolate mofetil, and prednisolone) for cardiac recipients with Marfan's syndrome as compared with similar doses in normal recipients [11]. As there are no reports for kidney transplantation, we should administer these drugs carefully. For our patient, we administered a dose of 4 mg/day methylprednisolone from day eight after transplantation. One animal study showed that administration of tacrolimus does not appear to exacerbate graft arterial aneurysm [12]. But this was only a 30-day followup, so further study should be aimed at confirming whether administration of tacrolimus and mycophenolate mofetil to patients with Marfan's syndrome contributes to the exacerbation of aortic aneurysm.

After cardiac transplantation, the incidence of aortic dissection in patients with Marfan's syndrome is about 30%–40% [13]. Some medications may slow expansion of the vessels. Until now, *β*-receptor antagonists were thought to cause exacerbation of the ascending aorta, but recently it was reported that angiotensin receptor blockers result in a significant reduction of the rate of change in aortic root diameter as compared with *β*-blocker therapy [14, 15]. Beta-blockade may reduce the onset of graft problems and new arterial aneurysms. 

We revealed that a living-related kidney transplantation to a patient of Marfan's syndrome was successful. However, the guidelines recommend taking a CT scan or MRI periodically [16], and there is a report about a case of a second retrograde common iliac artery dissection in a Marfan's patient after delivery of her child [17]. For this reason, we suggest that patients with Marfan's syndrome receive a CT scan or MRI at least once a year. Two years after transplantation, graft function remains normal, there is no rejection, and abdominal CT scan shows no exacerbation of an aneurysm. However, there are some unclear points regarding complications and graft survival. Because cases of Marfan's syndrome with advanced iliac artery dissection are rare, we need to evaluate and follow up more carefully transplant patients with Marfan's syndrome.

## Abbreviations

ESRD: End-stage renal disease
HLA: Human leukocyte antigen
TGF*β*: Transforming growth factor *β*.
